# Solo Aging In New England: Findings from the 2025 Healthy Aging Data Report

**DOI:** 10.1093/geroni/igaf122.2727

**Published:** 2025-12-31

**Authors:** Sophia Casale, Mengshi Liu, Michelle Ward, Dongfang Hong, Elizabeth Dugan

**Affiliations:** University of Massachusetts Boston, Boston, Massachusetts, United States; University of Massachusetts Boston, Boston, Massachusetts, United States; University of Massachusetts Boston, Boston, Massachusetts, United States; University of Massachusetts Boston, Boston, Massachusetts, United States; University of Massachusetts Boston, Boston, Massachusetts, United States

## Abstract

Experiencing later adulthood as a solo ager is gaining increasing research interest and adults who have never married are a major component of solo agers. This research describes community rates of adults 65+ who have never been married, the percentage of older adults who have annual incomes below the poverty level, and community medical service utilization rates (ER visits, and annual physician visits) in CT, MA, and RI. Data were from the 2025 Healthy Aging Data Reports. The indicators calculated community and state rates derived from the American Community Survey (2018-2022), and Centers for Medicare & Medicaid Services (2020-20221) data. Results showed that communities with highest percentage of never-married adults 65+ and the % of adults 65+ living below the federal poverty line are concentrated in urban areas: Hartford, CT (22.90%), Longwood (Boston), MA (57.69%) and Providence, RI (22.22%). Some of the lowest annual physician visit rates and highest annual ER visit rates are in urban/low-income communities with above state average never married 65+. Annual physician visits / year are low in Hartford, CT (6.18), Central Falls, RI (4.81), Providence, RI (6.68). Annual ER Visits / 1,000 adults 65+ are high in Hartford, CT (1,062.65), 6 Corners (Springfield), MA (1,025.32), East Boston, MA (868.89), Downtown (Worcester), MA (863.08), Chelsea, MA (822.52) and Woonsocket, RI (601.08). These findings provide important descriptive information about a group that tends to get overlooked. The findings capture evident disparities and may guide practitioners and policymakers in allocating resources to better serve the never-married 65+ population.

